# Roads and livelihood activity choices in the Greater Serengeti Ecosystem, Tanzania

**DOI:** 10.1371/journal.pone.0213089

**Published:** 2019-03-08

**Authors:** Solomon Zena Walelign, Martin Reinhardt Nielsen, Jette Bredahl Jacobsen

**Affiliations:** 1 Department of Food and Resource Economics, University of Copenhagen, Frederiksberg C, Denmark; 2 School of Economics, University of Gondar, Gondar, Ethiopia; 3 Center for Macroecology, Evolution and Climate, University of Copenhagen, Copenhagen Ø, Denmark; Chinhoyi University of Technology, ZIMBABWE

## Abstract

Road development is occurring at an unprecedented rate in important conservation areas in tropical countries with limited understanding of how local people will adjust their livelihood activities in response. We use a discrete choice experiment to explore the effect of road development on respondents *ex-ante* preferences for changes in livelihood activities—crop and livestock production, hunting and trading bushmeat, and business and wage employment—under different incentives—provision of loans, livestock and crop extension services–in scenarios with reduced travel time to nearest district town in the Greater Serengeti Ecosystem in Tanzania. We test four hypotheses about the effects of roads with opposing implication for conservation. Hypothesis 1 predicts that increased market access will lead to intensification of crop and livestock production activities (achieved through extension services and loans), and Hypothesis 2 that market access will facilitate the development of non-farm Micro, Small and Medium Enterprises (MSME) providing new livelihood opportunities (e.g. business income and wage employment)–both reducing environmental pressure. Hypotheis 3 on the other hand predicts that improved market access will lead to extensification and expansion of crop and livestock production activities, while Hypotheis 4 suggests that it will encourage exploitation of environmental goods (here in the form of hunting and trading bushmeat and illegal grazing inside protected areas)–both increasing environmental pressure. We find increasing preferences for more cropland and more cattle as travel time to market is reduced but no preference for increased allocation of household members to hunting and trading bushmeat supporting hypothesis 3 while contradicting hypothesis 4. However, second-order effects might support hypothesis 4 as we find aversion towards decreasing effort invested in hunting and trading bushmeat. Preferences for increased cropland and livestock may furthermore interact to increase land use change and illegal grazing inside protected areas. Crop extension services had a negative modifying effect on preferences for more cropland (supporting hypothesis 1) while livestock extension services had a positive modifying effect on preferences for more cattle (contradicting hypothesis 1). Providing loans had a negative modifying effect on preferences for increasing cropland and number of cattle. Marginal rates of substitution suggest that 950,000 TSH borrowed at a 10% interest rate will reduce preferences for more cropland and cattle by 11.8 and 38.4% respectively. Crop extension services reduce preferences for more cropland by 27% whereas livestock extension services increase preferences for more cattle by 104%. Contradicting Hypothesis 2, we found no preference for increasing the number of households members engaged in business and wage employment in response to reduced travel time. Targeted efforts to increase the educational level as well as entrepreneurship skills in the GSE could promote engagement in the labour market and development of business enterprises diverting focus from traditional activities such as farming and livestock production and hence reducing pressure on the ecosystem.

## 1. Introduction

Rural households in developing countries rely on surrounding ecosystems to provide a variety of services (incl. water, firewood, timber, medicine, grazing, and wild food) essential to sustain their livelihoods [1–3]). Growing human populations, expected to quadruple this century [4], and their livestock in communities adjacent to protected areas increase the pressure on environmental resources, negatively affecting conservation objectives [5–10]. The poor tend to rely more on ecosystem services [1,11] and may thus suffer disproportionate deprivation from depletion of environmental resources. Infrastructural development extending roads into remote rural areas is proposed to reduce poverty by facilitating market integration and growth of non-farm Micro, Small and Medium Enterprises (MSMEs) [12]. Consequently, road expansion is occurring at an unprecedented pace in developing countries extending into remote areas harbouring ecosystems with high biodiversity conservation value [13]. However, according to some observer’s risks are often not adequately assessed and roads may instead cause environmental, economic and socio-political problems [13–16].

In Tanzania, the proposed 548 km Northern Serengeti all-whether road aiming to connect the towns of Mto wa Mbu, Waso, Mugumu and Musoma in Mara and Arusha regions but also bisecting the World Heritage Site listed Serengeti National Park has sparked considerable debate [17–20]). Opponents suggest the road could disrupt the annual migration of ~1.5 million wildebeest, zebras and gazelles between Serengeti National Park and Masai Mara National Reserve in Kenya, increase poaching and reducing the tourist income potential among other consequences [19,21]. A study estimates a drop in the wildebeest population by 35% assuming the road will constitute a barrier to migration [22], which on the other hand has been contested [17]. Proponents argue that the road will facilitate national and local economic growth reducing poverty and improving the local quality of life [17,18], which is generally expected to lower pressure on ecosystems [23,24]. A study of the socio-ecological feasibility of two alternatives to the Northern Serengeti all-whether road, one of which passes along the southern edge outside the Serengeti National park found that the Northern Serengeti all-whether road was associated with least improvement in children access to schools, fewest households with increased access to hospitals, least connection of labour force with small and medium-sized business and between markets and areas with high crop and livestock production [25]. The Northern Serengeti all-whether road furthermore had the highest establishment costs and most negative impacts on conservation and tourism income potential. However, [23], retorts that the improved social well-being expected from the Northern Serengeti all-whether road is a human right and a prerequisite for conservation because the adjacent communities are the custodians of migratory species. With the lines thus drawn up, it remains unclear how communities will adjust their livelihood activities in response to road improvement in general, reducing travel time to markets. Such information is needed to facilitate informed predictions about emerging and changing environmental pressure as a result of land use change, overgrazing and hunting and bushmeat trade.

General economic theory and empirical findings suggest diverging effects of roads that have opposing implication for conservation [26]. We identify four simplified hypotheses based on the literature. Hypothesis 1 predicts that road improvement through increased markets access will enable people to earn a higher profit from their cattle and crops, leading to intensification of such activities [27–29] facilitated by livestock and crop extension services and loans. Hypothesis 2 furthermore propose that market access will facilitate the development of non-farm MSME providing new livelihood opportunities (e.g. business income and wage employment) [28,29]. Consequently, ecosystem pressure will initially decrease and biodiversity conservation will improve [30,31]. Hypothesis 3 suggests on the contrary that increased market access will lead to extensification, adding more land and stock to the production, which in this case may involve expansion of agriculture through landuse change and to illegal grazing of the increased livestock in protected areas increasing ecosystem pressure [32,33]. Note that intensification and extensification are not mutually exclusive and that both may require loans due to barriers to investment [34]. Hypothesis 4 similarly suggests that roads will increase extraction of resources from the environment due to improved access to protected areas exposing desirable environmental resources to urban market demand encouraging commercial larger-scale extraction by local people as they now can sell environmental goods at higher prices [35–38]. Commercialisation can arguably lead to overharvesting and depletion of ecosystem services [7,39]. Which of the four outcomes will prevail can as mentioned be influenced by initiatives assisting households to overcome barriers to investment and increased production such as loans and extension services. These considerations suggest that pre-implementation evaluation of the likely effect of infrastructure development on households choice of livelihood strategy is necessary to mitigate unintended consequences.

This study, therefore, aims to (i) assess the effect of potential road development across the Greater Serengeti Ecosystem (GSE) on households’ preferred livelihood activities in effect indirectly testing the support for the four hypotheses (cf. above) and (ii) explore heterogeneities in preferences across socio-economic covariates. Effects of such changes can typically only be observed ex-post, preventing the design of timely mitigating policies and strategies. Consequently, we apply a discrete choice experiment to evaluate people’s expected adjustment in livelihood activities and to test the described hypothesis we focus on change in number of livestock and cropland (Hypothesis 1 vs. 3), household members allocated to business and wage employment (Hypothesis 2) and to hunting and trading bushmeat (Hypothesis 4), as an effect of decreased travel time to nearest district town due to hypothetical road construction or upgrading of existing roads across the GSE and how this effect is modified by the provision of loans and extension services (which also reflects on Hypothesis 1). Hence, this paper speaks broadly to the development and conservation literature concerned with the current explosion of infrastructure in developing countries and its impact.

## 2. Method

### 2.1. Study area

**Fig 1:**
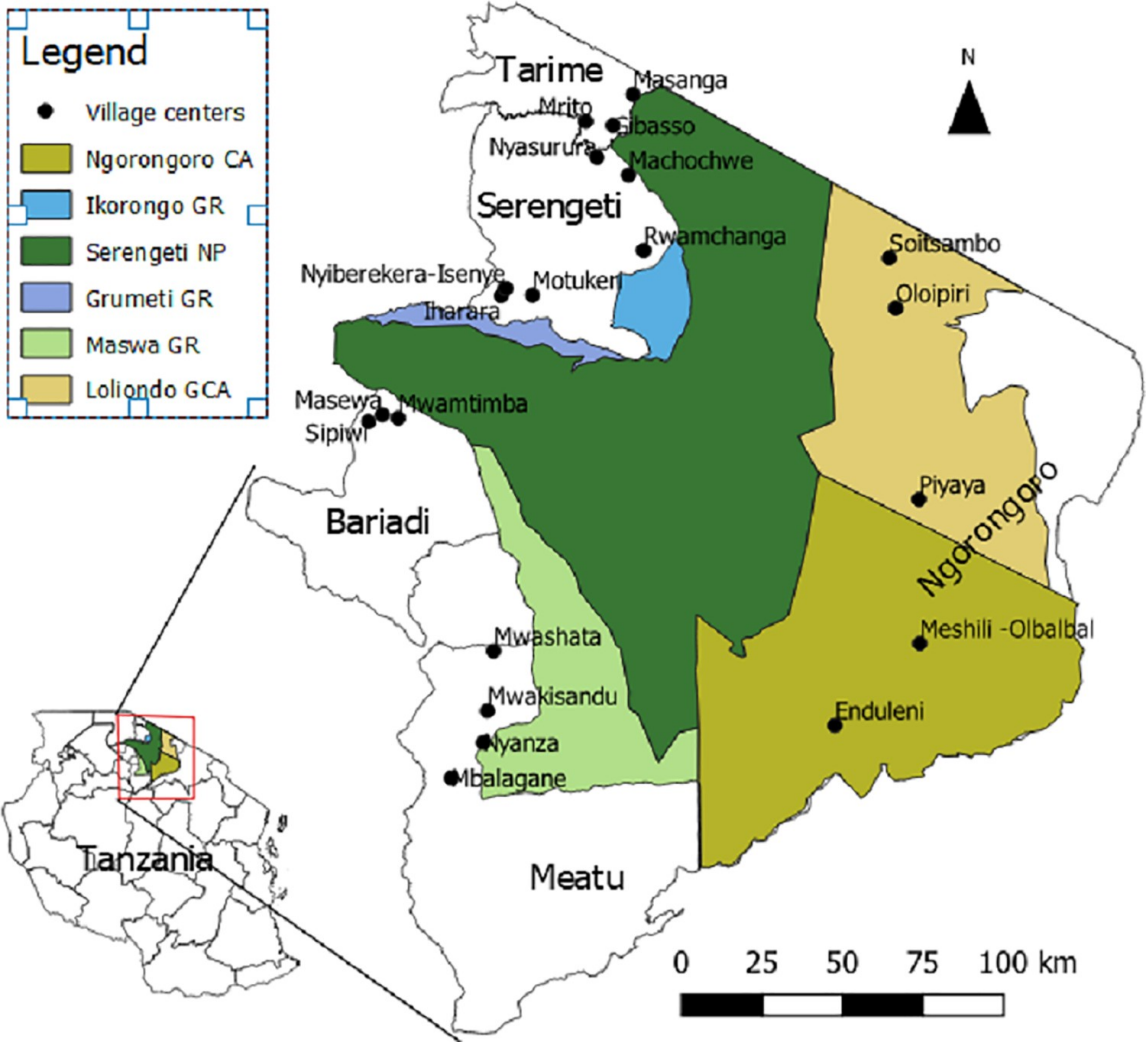
Surveyed villages and protected areas in the Greater Serengeti Ecosystem. CA = Conservation area; GR = Game Reserve; NP = National Park; and GCA = Game Controlled Area.

Data was collected in the GSE covering an area of about 18,000 km^2^ in Tanzania on the border of Kenya. The topography of the ecosystem is dominated by plains hosting the greatest remaining wildlife migration in the world following seasonal variations in rainfall and the availability of grazing across the ecosystem [40]. Outside the protected areas’ boundaries lie agricultural and pastoral areas home to over two million people in the nearest seven districts [41]. The Maasai inhabit the Loliondo Game Controlled Area and the Ngorongoro Conservation Area on the eastern boundary of the Serengeti National Park stretching to the southern edge of the plains where they meet the Sukuma agropastoralists as the principal inhabitants of the area South and South-West of the Maswa Game Reserve up to Lake Victoria. North of the western corridor and West of Ikoma, Ikorongo and Grumeti Game reserve is densely populated agricultural land mainly inhabited by the Ikoma and the Kuria tribe further North. The human population in the ecosystem is growing at an alarming pace of 3.5% per year increasing the pressure on the ecosystem to meet demand for food, energy, construction material, water, and land with conversion of natural habitats to agriculture at a rate of 2.3% per annum [42,43]. Conversion of land to agriculture is fastest along the western border of the park where human population growth rates are also highest. Although agriculture is increasing in the pastoralist areas east of the park, land-conversion is still minimal. Overall land use change appears driven by frontier engulfment by people being pushed towards park boundaries by resource scarcity in distant densely populated areas [44,45]. Living close to the protected areas is characterised high levels of human-wildlife conflict lowering agricultural outputs [46] and higher levels of poverty and poor health [47]. Poverty is prevalent around the Serengeti ecosystem with per capita income below the average national and ~75% of households living below the basic needs poverty line for Tanzania of 0.76 US per day [48]. Poverty appears to be a key driver of bushmeat hunting in the western and southern part of the ecosystem mainly for income generation through the bushmeat trade [49,50]. Estimates of the number of people hunting in the protected areas vary considerably from 9–32% of the population [51] up to 52,000 people in Western Serengeti alone [50]. The intensity of hunting is expected to increase [52] because of the rising human population adjacent to the protected areas [53]. An estimated 20% of households furthermore illegally graze livestock inside the protected areas [54]. Hence, the GSE was selected for this survey because of the proposed infrastructure project but also due to the increasing pressure on ecosystems in this social-ecological system. The proposed Northern all-weather road involves upgrading an existing gravel road to a tarmac road connecting the Mara and Arusha regions and particularly the headquarter of Serengeti district, Mugumu, and the headquarter of Ngorongoro district, Waso. However, we take a broader look at preferences for livelihood activities in response to road development considering both construction of new roads and upgrading of existing roads across the GSE. Study villages were selected in Ngorongoro, Meatu, Bariadi, Serengeti, and Tarime districts (Fig 1).

### 2.2. Data collection

Households, defined as a group of people sharing food, income and labour, were selected using a three-stage stratified sampling strategy. In the first stage, 21 villages were purposively selected in clusters of three in the five districts adjacent to the protected areas aiming to provide variation in distance to the boundary, ethnic composition and dominant livelihood strategy, precipitation and soil quality, habitat and infrastructure (Fig 1). The districts differ markedly in precipitation, soil characteristics, human population density, ethnic composition and level of development (Table 1).

**Table 1:** Socio-economic and biophysical characteristics of study districts.

District	Rainfall (average annual between 1983–2010) (mm)	Temperature (average annual between 1961–2014) (°C) [min, max]	Soil Type	Population density (# per square kilometre)	Major ethnic group	Dominant livelihood strategies	Level of development (i.e. roads and market access)	Total villages (rural and urban)
Tarime	98	[16, 30]	Luvic Phaeozems/ Euthric Leptosols	77	Kuria	Farming/ non-farm wage	Higher	108
Serengeti	78	[16, 29]	Luvic Phaeozems	25	Kuria/ mixed	Farming/ Pastoralism	Higher	94
Bariadi	67	[16, 29]	Mollic Solonetz/ Eutric Planosols	29	Sukuma	Farming/ Pastoralism	Higher	113
Meatu	59	[16, 29]	Chromic Cambisol/ Mollic Andosols	36	Sukuma	Pastoralism / Farming	Lower	99
Ngorongoro	58	[13, 26]	Chernozems	14	Massai	Pastoralism	Lower	40

Source: Global gridded soil information (http://www.isric.org/explore/soilgrids), GRWv4 gridded population datasets (http://beta.sedac.ciesin.columbia.edu/data/set/gpw-v4-population-count-adjusted-to-2015-unwpp-country-totals), Tanzania Population Census 2012 (http://www.nbs.go.tz/) and Tanzania Meteorological Agency (http://maproom.meteo.go.tz/maproom/).

In the second stage, 40 households were selected in each village using a stratified random sampling based on participatory wealth ranking generating a sample of 840 households (sampling intensity ranges between 3.4 and 16.5%. Wealth ranking was undertaken by a group of knowledgeable village members based on locally agreed criteria, following [55]. All households in each village were placed in one of three wealth categories—rich, intermediate and poor—and 10, 20, and 10 households respectively were selected randomly from these categories using a random number generator in Excel. All households in this sample were subjected to a questionnaire survey aiming to capture information on cash and subsistence income from all livelihood activities as well as development in assets through four quarterly interviews over the course of a year applying the Poverty-Environment Network (PEN) survey protocol (see [56] and Appendix A in S1 File). The PEN approach involves four quarterly surveys over the course of a year to increase data accuracy by capturing seasonal variation in income (e.g. crop income) and minimising recall bias by reducing the recall period (1–3 months depending on income source). In the third stage, a sub-sample of half of the households in each wealth group within a village (i.e. about 19–20 households in each village) were randomly selected and subjected to the choice experiment. Chosing to use this subsample was based on budget constraints, other research objectives and because this sample would be sufficient to answer research questions. One village located on a tarmacked road close to the district centre was excluded from the sample because travel-time reduction scenarios would not be credible or relevant in this village. Hence, the final sample for the choice experiment contained 381 households. In addition, focus group discussions (FGD) and a pilot survey were conducted with households not included in this sample contributing towards development of the choice experiment survey (see section 2.3 for details).

### 2.3. Choice experiment design

Choice experiments is a stated preference method with more than 20 years of use in developing countries [57]. It allows elicitation of comparable measures of preferences across factors and attributes and is increasingly used to asses resource users preferences in the context of conservation due to its (i) relevance allowing evaluation of preferences in relation to possible policy changes [58–60] and (ii) its ability to elicit information about sensitive livelihood activities, such as bushmeat hunting [61–63]. A choice experiment design was developed to evaluate likely changes in livelihood activities in response to the construction of new roads, or upgrading of existing roads reducing travel time to the nearest district market by 10, 25 or 50%. The choice experiment asked the household head to pick the preferred of three alternative livelihood options in different scenarios of travel time reduction. Each alternative is described by a set of attributes reflecting prevalent livelihood activities in the GSE. Carrying out several such choices in scenarios varying in the level of the attributes allow systematic evaluation of preferences.

The choice experiment design was developed based on qualitative and quantitative pre-testing to establish the credibility and acceptance of the baseline condition, the mechanism of change, the change to be valued and the payment vehicle by respondents through FGDs in accordance with state-of-the-art guidelines [64]. Hence, development of the design involved four steps. First, the Sustainable Livelihoods Framework (SLF) [65,66] was used as a conceptual basis for selecting attributes. The SLF argues that the choice of livelihood strategy is a function of households’ assets, abilities and context. It focuses on five capital assets–natural, physical, human, social and financial assets–that can be stored, accumulated, exchanged and allocated to generate a flow of income through market transactions and subsistence production upon which livelihoods are built. A list of attributes potentially relevant to describing the change in rural livelihood activities was developed by reviewing empirical studies using the SLF.

Second, this list was presented to focus groups, and the relevance of each item explored. FGDs were conducted in the study villages Gibaso, Issenye, Oloipiri and Mwantiba resulting in the selection of seven attributes. The attributes were assigned levels based on the FGDs (Table 2). Choice-cards were developed with three alternatives each. Two alternatives represent scenarios of changing livelihoods while the third represents a ‘status quo’ option to avoid forced spurious choices for households unwilling to trade the current situation against any of the alternatives [67]. All attributes varied across alternatives, except travel time reduction that was fixed within choice-sets to reduce the cognitive burden on respondents.

**Table 2:** List of attributes and their level in the choice experiment.

Attribute	Description	Level	Hypothesis
Wage employment change	Change in number of adult household members engaged in full-time employment	Decrease of one, no change (reference level), and increase of one	Reduced travel time increases preference for wage employment as it increases access to labour markets
Land use change	Additional area of land allocated to crop production (including cash crops) through conversion of land currently under other use	0% (reference level), 10%, and 50%	Reduced travel time increases preference for higher proportion of land allocated to crop production
Change in cattle	Change in number of cattle owned (in percentages)	-50%, 0% (reference level), and 25%	Reduced travel time increase preference for larger cattle stock because it provides access to markets for meat and milk.
Extension services	Provision of improved seeds and veterinary services	No extension service (reference level), agricultural extension service, and livestock extension service	Extension services modify preferences increasing the preference for increased land conversion to agriculture and more livestock as an effect of reduced travel time.
Change in hunting and trading bushmeat	Effort hunting and trading bushmeat relative to today	Increase, no change (reference level), and decrease	Reduced travel time increases the opportunity costs of hunting reducing preferences for hunting and trading bushmeat
Loan	Magnitude of a loan (in Tanzanian Shillings) at a 10% interest rate repaid in four instalments within a year	0 (reference level), 50, 200, 500, 1000, and 3000 (thousands)	Reduced travel time creates preference for higher loans as the profitability of investments increases with improved market access.
Reduction in travel time	Reduction in travel time to the nearest district town due to road connectivity improvement.	0% (reference level), 10%, 20% and 50%	Reduced travel time influences choice of livelihood activities

Third, the design was tested in 64 randomly selected households in the same four pilot villages (cf. above) to evaluate and enable adjustment of the survey tool and to generate priors for the final design. A d-efficient design was developed in NGENE using as priors estimates from a multinomial logit model (MLM) based on the data from the pilot survey in the 64 households. The design had the constraint that travel time was the same across the three alternatives in a choice-set. Based on this design twelve choice-sets were generated and distributed into two blocks (ex-ante d-error of 0.005).

**Fig 2:**
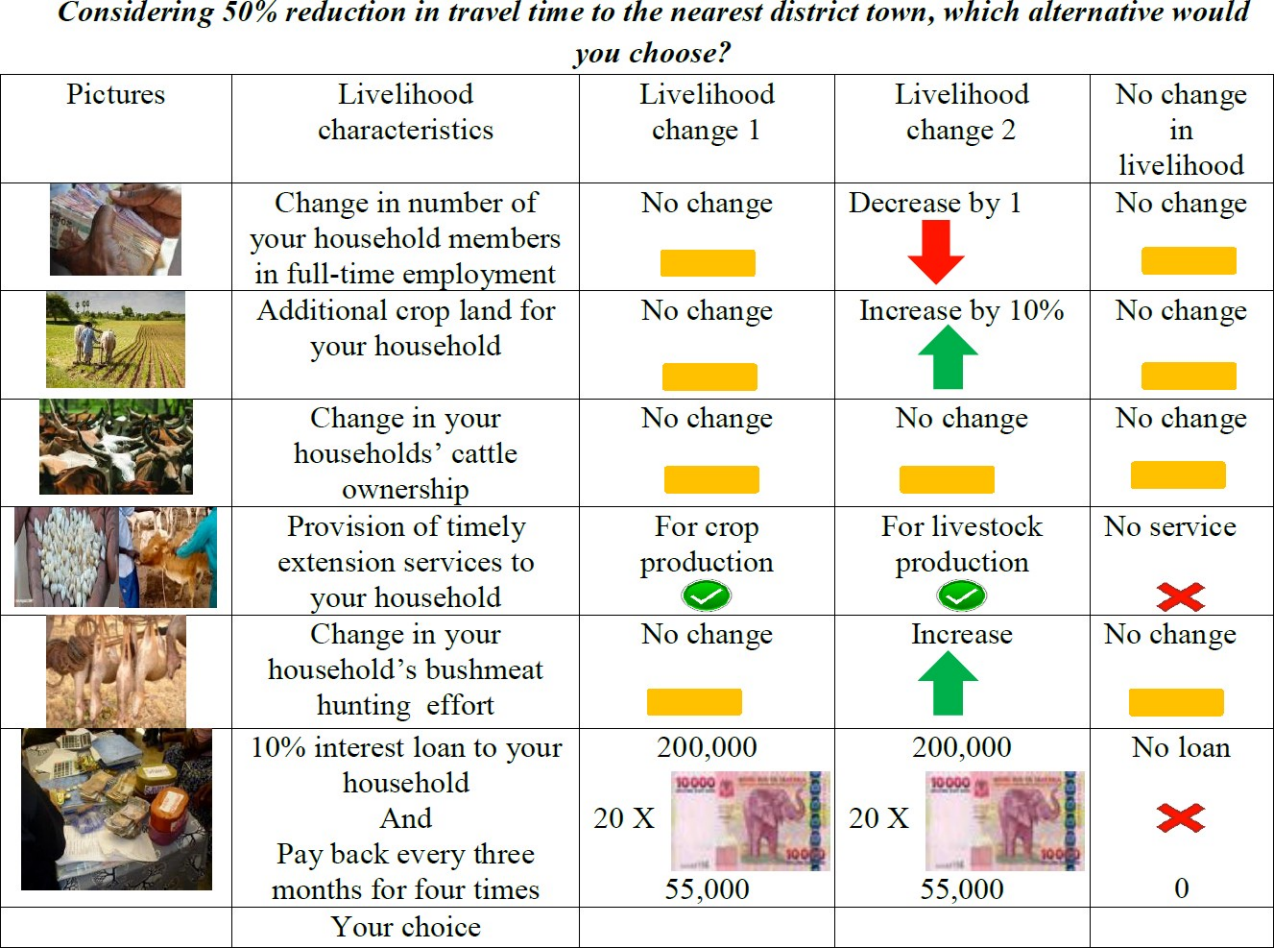
Example of a choice card in the choice experiment design. From top to bottom; the pictures portray a payment made in wage employment or a business transaction, agriculture, livestock, provision of improved seeds as an example of agricultural extension services, medical service to livestock as an example of livestock extension services, wild animals killed by bushmeat hunters for consumption and trade, and saving and credit service in rural areas, respectively.

Fourth, choice-sets were transformed into a choice experiment questionnaire including an introductory text for enumerators to explain about the objective of the study and the definition of the attributes in the choice experiment and their levels (see Appendix C in S1 File, for exact translated wording). A cheap talk script was included to minimise bias arising from the hypothetical nature of the experiments [68]. The script also included a reminder urging respondents to consider cost, benefits and associated risks to household welfare. This included informing respondents that choosing one strategy meant that they would have fewer resources available to adopt or continue other livelihood activities simultaneously. Consequentiality was established by specifying that the study might be used for policy design. Illustrative pictograms of attributes and signs indicating the direction of change of attribute levels were included to facilitate illiterate respondents understanding and reduce cognitive burden (see an example of a choice card in Fig 2). Households was presented with a random block, and each household conducted six choice tasks. Choices were recorded using Open Data Kit (ODK) through a tablet interface which also enabled showing pictures and videos illustrating the attributes to the respondents on the tablet while explaining the experiment. “Percentages” were also explained this way to respondents unfamiliar with the concept (Appendix B in S1 File). Follow-up questions were included to identify irrational players defined here as individuals who objected to the scenario and did not strive to maximise own utility (n = 0).

### 2.4. Ethics statement

This study passed an EU Horizon 2020 ethics review procedure before commencement of the project (proposal number 641918). The ethics advisory group of the Department of Food and Resource Economics at the University of Copenhagen evaluated and approved the ethics guidelines, and the free prior informed consent form and the survey tools developed and used during the study and the procedures applied to ensure respondent confidentiality and anonymity (ID: GA641918). As the data was collected using tablets and due to low level of literacy in the study area, the consent statement was read by the enumerators to the respondent, and the consent for participation was obtained verbally and entered to the tablet by the enumerator. The Tanzanian Commission for Science and Technology granted permission for implementation of the study (research permit No 2017-299-NA2011-21) involving an initial assessment of the objectives and ethical procedures of the project. Finally, procedures for collecting information from human subjects were approved by the Tanzanian National Health Research Ethics Committee (ID: NIMR/HQ/R.8a/Vol. IX/2609).

### 2.5. Data analysis

Analysis of the choice experiment takes departure in random utility theory, which posits that individuals when making choices, choose the alternative that yields the highest benefit, i.e. utility (see Appendix D in S1 File). The level of utility is defined by the level of the attributes in the chosen alternative plus a random component, unobservable to the analyst [69]. Different modelling options exist to estimate parameters. We used a random parameter logit model (RPL) preferred for its computational flexibility in modelling preference heterogeneity and in relaxing the assumption of Independence of Irrelevant Alternatives (IIA) [70]. Following [70], the random parameter logit model can be specified in terms of the probability of individual *n* choosing alternative *i* as the integral of the conditional logit probabilities over a density of parameters:

$$P_{ni}=\int [(\exp(\beta_{n}^{'}X_{ni})/\sum_{j}exp(\beta_{n}^{'}X_{nj})]f(\beta )d\beta$$ (1)

where *P*_*ni*_ is the choice probability under the random parameters logit model, β’_n_ is a vector of parameters for the vector of attributes X_*ni*_ of the *j*’th alternative presented to the respondent (see Table 2 for the list of attributes). To capture preference heterogeneity, the coefficients were allowed to vary over households in the population with density *f*(*β*), which is the function of parameter *θ* that represents the distribution parameters of *β*_*n*_ (in this case the mean).

The effect of the road was specified in the choice experiment as the percentage reduction in travel time and recalculated as the actual time spend post road implementation based on the average stated time spent travelling during the dry and the wet season to the nearest district town. As we aimed to analyse the response of people’s livelihood choices to travel time reduction, we interact travel time reduction with the attributes in the model. To capture that a relative decrease in the level of attributes representing livelihood activities that individual households do not engage in is not applicable, the attribute levels were changed to zero at the analysis stage for relevant households determined through socioeconomic information based on the PEN survey (cf. above). In total 600, 1248, and 1478 changes were made at the analysis stage for cattle, wage and business employment, and effort hunting and trading bushmeat, respectively of a total of 6858 choices made). These changes correspond to the instructions given to respondents to ignore irrelevant attributes (i.e. attribute non-attendance) in the scenario description. A fixed alternative specific constant (ASC) was included in the model representing the status quo alternative.

Heterogeneity was allowed on all attributes, except loan that was fixed to estimate the marginal rate of substitution for other attributes. However, we controlled for potential preference heterogeneity for loan through its interaction with income. The term of this interaction was insignificant, and the interaction was hence excluded from the final model. The model was estimated with 2000 Halton draws using the mlogit and gmnl packages in R [71,72].

The proportion of the sample with a positive preference for individual random attributes (*p*_*i*_) was estimated using the following equation:

$$p_{i}=\phi \left(\frac{\beta_{i}}{\sigma_{i}}\right)$$ (2)

where *ϕ* is the cumulative standard normal distribution, *β*_*i*_ and *σ*_*i*_ are the mean coefficient and standard deviation of coefficient of the individual random attribute *i*. The marginal rate of substitution between loan and the rest of the attributes ($MRS_{\beta_{i}\beta_{l}}$) was estimated based on the equation:

$$MRS_{\beta_{i}\beta_{l}}=\left(\frac{\beta_{i}}{\beta_{l}}\right)$$ (3)

where *β*_*i*_ and *β*_*l*_ are the coefficients for attributes *i* and loan. Standard errors were estimated using the delta method. Following [59], the MRS was recalculated to Willingness To Pay (WTP) assuming that an alternative loan would have an interest of 50% based on information from the FGDs, using the equation:

$$WTP_{\beta_{i}\beta_{l}}=MRS_{\beta_{i}\beta_{l}}*VSI$$ (4)

where $WTP_{\beta_{i}\beta_{l}}$ is the marginal rate of substitution for attributes *i*, and *VSI* is the value of the saved interest, which can be calculated as the difference between the alternative loan and the loan in the choice experiment considering the four annual payments (i.e., (1+0.5/4)^4^−(1+0.1/4)^4^ = 0.497994). To explore preference heterogeneity further, we extracted households’ posterior β’s for attributes with significant heterogeneity in the RPL and estimated these as a function of selected socio-economic variables from the PEN survey (cf. above) using a linear mixed model with district ID as a random intercept [73]. The choice of which socio-economic covariates from the PEN survey to include was motivated by general economic theory and empirical evidence form previous studies (see Appendix E in S1 File).

## 3. Results

### 3.1. Preference for change

The coefficients of the RPL are presented in Table 3. Most attributes have significant effects although McFadden’s pseudo-R-squared is relatively low (pseudo-R^2^ = 0.08). The coefficients represent the mean change in utility for one unit change in the level of that attribute holding all other attributes constant. The ASC×travel time reduction interaction is significant and negative indicating that respondents on average preferred changing their livelihood over maintaining status quo when travel time is reduced. For an average household, the travel time reduction was 27 minutes (the average distance of sample households to the nearest district town). Preference for increase of cropland and number of cattle increased significantly with increasing travel time reduction. Larger loans, and crop and livestock extension services were also preferred significantly more the larger the reduction in travel time. Preference for reducing the number of household members engaged in business and wage employment decreased significantly whereas preference for increasing the number of household members hunting and trading bushmeat decrease significantly in response to reduced travel time. Coefficients for increased business and wage employment and decreased effort hunting and trading bushmeat were statistically insignificant.

**Table 3:** Coefficients of the Random Parameter Logit model (RPL) indicating the mean respondent preference for livelihood activities and incentives in the context of reduced travel time to nearest District town. Values in parenthesis are standard errors. Standard deviations are calculated based on the mean coefficients of the attributes that were set as random. The estimated percentage is the percentage of households that have the same sign as the mean coefficient for their preference (Eq 2). MRS is the marginal rate of substitution of the attributes with respect to loan (Eq 3). The associated standard errors were estimated using the delta method. WTP is the MRS recalculated to Willingness To Pay assuming that an alternative loan would have an interest of 50% (Eq 4).

	Mean coefficients	Standard deviations	Estimated percentage	MRS	WTP
Wage and business employment (decrease by one households member) × travel time reduction	-0.025*** (0.009)	0.115*** (0.027)	41.39	-1230780 (559475)	-612921
Wage and business employment (increase by one households member) × travel time reduction	-0.004 (0.010)	0.065*** (0.015)	47.30	-217645 (483557)	-108386
Increase in crop land (percentage/100) × travel time reduction	0.164*** (0.045)	0.047*** (0.017)	99.97	80432 (9042)	40055
Change in cattle ownership (percentage/100) × travel time reduction	0.050*** (0.014)	0.086*** (0.015)	71.98	24737 (4970)	12319
Extension services (crop production) × travel time reduction	0.044** (0.019)	0.137* (0.082)	62.45	2139152 (574742)	1065284
Extension services (livestock production) × travel time reduction	0.052*** (0.018)	0.037*** (0.009)	92.05	2535553 (665488	1262690
Decreased effort hunting and trading bushmeat × travel time reduction	-0.005 (0.060)	0.118*** (0.017)	48.47	-221701 (2936048)	-110406
Increases effort hunting and trading bushmeat × travel time reduction	-0.010** (0.005)	0.150*** (0.023)	47.46	-470122 (251267)	-234118
Loan/1000000 × travel time reduction	0.020*** (0.005)	-	-	-	-
ASC: status quo × travel time reduction	-0.205*** (0.027)	-	-	-10065011 (3329690)	-5012313
Log Likelihood	-1778.4				
McFadden Pseudo R-squared	0.082				
# of observations	2286				
# of respondents	381				

***, ** and * represents significance at the 0.01, 0.05 and 0.1 level.

The standard deviations for all random attributes reveal that there is significant heterogeneity in respondents’ preference for all attributes. Hence, we cannot rule out that the insignificant mean parameters for increased business and wage employment and decreased effort hunting and trading bushmeat influence the utility of some respondents. To explore this heterogeneity, we calculated the proportion of the sample with preference for each attribute based on the magnitude of the standard deviations relative to the mean coefficients (Eq 2). If travel time is reduced, almost all households (~99%) would according to their choices convert to more cropland, and about ~72% would opt for more cattle. Preferences are more divided with respect to business and wage employment where ~41% would choose a decrease, and ~47% prefer an increase (compared to no change). Similarly, ~48% prefer decreased effort allocated to hunting and trading bushmeat while ~47% preferred increased effort. Approximately 62% and ~92% prefer provision of crop and livestock extension services respectively (compared to no extension services) (see Table 3).

An alternative model with interactions between selected activity attributes and incentive attributes—i.e. between increase in cropland, number of cattle and wage employment and attributes reflecting potential management and policy tools, namely low-interest loans and crop and livestock extension services—reveals (i) a negative modifying effect of both crop extension service and loan on increase in cropland, (ii) a negative modifying effect of loan and a positive modifying effect of livestock extension services on change in cattle numbers and (ii) a positive modifying effect of loan on increased wage employment (see Appendix H in S1 File). This suggests that incentives for some activities may be modified through these policy tools.

### 3.2. Marginal rates of substitution

The results of respondents trade-off rate–i.e. Marginal Rate of Substitution (MRS)—presented in Table 3, was calculated based on the ratio between the mean coefficients of an attribute and a loan, thus representing the amount of loan the households would be willing to sacrifice for the benefit of one unit of another attribute while maintaining their utility (here well-being) at the same level. The utility of 1% increase in cropland and the number of cattle owned is equivalent to the utility of a loan of 80,432 and 24,737 TSH at a 10% interest rate, respectively. In other words, households on average would trade-off or sacrifice an 11.8 or 38.4% increase in cropland or cattle respectively for a loan of 950,000 TSH roughly equivalent to the average annual income per AEU. However, the value of the specified loan depends on alternative loan options available. FGD’s revealed that available loans varied considerably in terms of annual interest rates (from 20% to 100%) and number of instalments (every week to once per year), thereby also in the absolute value of such loans. If the alternative available loan has an interest rate of 50% (common in pilot test villages), the value of the saved interest is 0.498% (see Eq 4). Hence, the value of a loan of 80,423 TSH (equivalent to the utility of a 1% increase in cropland) is 40,055 TSH, and a loan of 24,737 TSH (the utility of 1% increase cattle) is 12,319 TSH. This is also called a Willingness To Pay (WTP) measure, reflecting the saved amount that an average household is willing to sacrifice. Similarly, the utility of an additional household member engaging in hunting and trading bushmeat is equivalent to the utility of a loan of 470,112 TSH in the scenario context. The provision of crop extension service is equivalent to an increase in cropland of 27% while the provision of livestock extension service is equivalent to a change in cattle numbers by 104%. The decrease of one household member in wage employment is traded-off for an increase in cropland and cattle of 15% and 50% respectively. An increase in effort hunting and trading bushmeat of 2.5 times is equivalent to wage employment by one household member.

### 3.3. Geographic and socio-economic variation

Preferences varied considerably between districts. In the full sample, preferences for increased cropland was approximately three times higher than an increase in the number of cattle. Crop extension service was less relevant compared to livestock extension service. However, preferences varied across districts with preferences for increased cropland only being three times higher than for cattle in Ngorongoro district compared to six times higher in Tarime district (estimation based on average posterior beta’s by district, Appendix F in S1 File). Crop extension service was preferred more in Meatu, Bariadi and Tarime districts compared to Ngorongoro and Serengeti. Preference for decrease in effort hunting and trading bushmeat was negative in Bariadi and Tarime while preference for decrease was positive in the three remaining districts. Preference for increased effort hunting and trading bushmeat was negative in all districts, except Meatu. Preference for decreased business and wage employment was negative in all districts, but preference for increased business and wage employment was also negative in Serengeti and Ngorongoro districts (Appendix F in S1 File). The prevailing interest rate for loans differed considerably between villages according to FGDs. This may cause heterogeneity between villages. However, as the coefficient of the interaction between loan and income was insignificant (Appendix I in S1 File), the effect of variation in interest rate is likely constant across villages and has little implication for differences observed here.

The effect of selected socioeconomic covariates on respondent preferences (marginal utilities) for the random parameters are explored in Table 4 using a linear mixed model with district-level random intercepts (see Appendix G in S1 File). Respondents from poor households had significant aversion towards increased effort hunting and trading bushmeat as well as towards business and wage employment but preference for increased cropland, cattle and crop extension services compared to respondents from households of intermediate wealth (the reference level) in the same district. Respondents from wealthy households on the other hand had significant preference for decreased effort hunting and trading bushmeat and business and wage employment. Compared to all other tribes than Kuria and Sukuma, Maasai respondents expressed significant aversion towards increased effort hunting and trading bushmeat and business and wage employment but preference for an increase in cropland and cattle and both livestock and crop extension services. Sukuma respondents, relative to other tribes than Kuria and Maasai, were averse towards livestock extension service but expressed a preference for increased business and wage employment. Similarly, Kuria respondents expressed aversion towards any change in effort hunting and trading bushmeat (i.e. more or fewer household members engaged in hunting) and towards decreased business and wage employment. The further away households were located from protected area boundaries—the more averse respondents were towards increased effort hunting and trading bushmeat and business and wage employment while favouring livestock and crop extension services.

**Table 4:** Linear mixed model with district-level random intercept for socioeconomic covariates of the posterior beta’s (marginal utilities) of parameters with significant heterogeneity (i.e. significant standard deviations in Table 3). Values in parenthesis are standard errors.

	Wage decrease	Wage increase	Increase in cropland	Change in Cattle	Livestock extension service	Crop extension service	Increased effort hunting and trading bushmeat	Decreased effort hunting and trading bushmeat
Household wealth ranking: Poor	-0.005 (0.004)	-0.016* (0.008)	0.022* (0.013)	0.007 (0.007)	0.026* (0.014)	0.019* (0.011)	-0.009** (0.004)	-0.017 (0.012)
Household wealth ranking: Rich	0.006** (0.002)	-0.017 (0.011)	0.006 (0.011)	0.007 (0.010)	0.030 (0.021)	0.023 (0.017)	-0.001 (0.004)	0.028*** (0.005)
Head tribe: Maasai	-0.001 (0.005)	-0.050*** (0.005)	0.031** (0.015)	0.044*** (0.005)	0.096*** (0.011)	0.083*** (0.009)	-0.014*** (0.004)	0.024** (0.009)
Head tribe: Sukuma	-0.004 (0.008)	0.009** (0.004)	-0.010 (0.014)	0.003 (0.003)	-0.018** (0.009)	-0.010 (0.008)	-0.001 (0.003)	-0.022 (0.015)
Head tribe: Kuria	-0.011*** (0.003)	0.009 (0.009)	0.018 (0.012)	-0.002 (0.005)	-0.011 (0.019)	-0.006 (0.015)	-0.005* (0.003)	-0.026* (0.015)
Total implements: Value	-0.004*** (0.000)	-0.002 (0.002)	0.008*** (0.001)	0.003** (0.001)	0.005** (0.003)	0.005** (0.002)	-0.003*** (0.001)	-0.010*** (0.002)
Total livestock: TLU	0.004*** (0.001)	-0.004 (0.005)	-0.004 (0.008)	0.000 (0.001)	0.002 (0.009)	-0.001 (0.006)	-0.002 (0.002)	-0.001 (0.003)
Total crop land: Acre	2.92X10^-4^ (0.004)	0.001 (0.010)	0.013 (0.010)	-0.008 (0.006)	0.005 (0.019)	0.001 (0.014)	0.003 (0.002)	0.015 (0.019)
Total grazing land: Acre	-0.003 (0.004)	-0.006 (0.004)	0.021 (0.016)	-0.003 (0.007)	0.014** (0.007)	0.009 (0.006)	-0.002 (0.003)	0.002 (0.010)
Distance to border of protected areas: Meters	1.69X10^-5^ (0.001)	-0.001** (4.21X10^-4^)	0.001* (3.88X10^-4^)	0.001 (4.42X10^-4^)	0.001** (0.001)	0.001** (0.001)	4.90X10^-4^* (2.91X10^-4^)	4.91X10^-4^ (0.002)
Constant	-0.004 (0.008)	0.038** (0.017)	0.098*** (0.019)	0.019 (0.012)	-0.027 (0.025)	-0.022 (0.020)	0.017** (0.008)	0.048** (0.021)
District: sd (constant)	0.004 (0.002)	1.85X10^-11^ (1.37X10^-13^)	3.80X10^-11^ (5.99X10^-9^)	2.45X10^-12^ (1.81X10^-11^)	1.55X10^-11^ (1.65X10^-9^)	2.74X10^-11^ (3.82X10^-9^)	2.06X10^-12^ (2.27X10^-10^)	0.012 (0.006)
District: sd (residual)	0.023 (0.001)	0.055 (0.004)	0.064 (0.007)	0.042 (0.002)	0.097 (0.008)	0.077 (0.006)	0.021 (0.002)	0.064 (0.005)
Log likelihood	895.33	564.38	508.05	670.54	347.37	434.53	933.13	502.01
# of groups	5							
# of respondents	381							

***, ** and * represents significance at the 0.01, 0.05 and 0.1 level.

## 4. Discussion

### 4.1. Livelihoods changes as a consequence of roads

We find that respondents in the GSE expect to make changes in livelihood activities in response to the construction of new and upgrading of existing roads depending on the reduction in travel time to the nearest District town with important conservation implications. Preferred changes involve in particular extensification, adding more cropland and also more cattle and the preference for these increase as travel time is reduced providing more support for Hypothesis 3 than Hypothesis 1, although these are not mutually exclusive. Hence, preferences for agricultural and livestock extension services also increased with reduced travel time indicating a desire for intensification of production supporting Hypothesis 1. Facilitating these changes preference for the amount borrowed in low-interest rate loans increased with reduced travel time. Further supporting the adverse environmental consequences of Hypothesis 3 the second order implications of preferences for more cropland and cattle may interact to increase pressure on the ecosystem. If reduced travel time to market drives further conversion of land to agriculture reducing already scarce grazing land in many locations, this may combine with the desire for more cattle to provide further impetus for illegal grazing of cattle inside protected areas. High grazing pressure inside the protected areas compresses habitat available to wildlife increasing competition for food and the risk of disease transmission [74].

Surprisingly, and contradicting Hypothesis 2, we found no preference for increasing the number of households members engaged in business and wage employment in response to reduced travel time. On the other hand, we found no preference for reducing labour market and business engagement either. The explanation may involve the generally low education level (88% of sample household members above 15 years of age had completed less than seven years of education) as well as low capacity for business development and entrepreneurship and financial constraints perceived as a barrier to further employment in occupations benefitting from reduced travel time to markets whether involving formal employment in district towns or MSMEs. Lack of skills is by community members perceived as a reason for low local employment in tourist camps in adjacent Wildlife Management Areas (WMAs) [75] and our extended model with interactions showed a significant positive modifying effect of loan (that relaxes the financial constraint) on the preference for increased business and wage employment (Appendix H in S1 File). The observed high preference for more cropland and cattle may also influence the perceived availability of household labour for wage employment.

We found evidence for aversion towards increased effort hunting and trading bushmeat and a insignificant preference for decreasing it. Hence, this result at a first look contradicts Hypothesis 4 suggesting that households at least do not expect to increase their involvement in hunting and trading bushmeat as market access improves and other options become more readily available. However, second-order and more long-term outcomes are unclear as respondents have no preference for reducing hunting and trading bushmeat either. Hence, as transport costs are reduced, and connection to higher purchasing power urban populations are improved focus on subsistence use, and local trade may shift to supplying urban markets in effect producing the scenario in Hypothesis 4. Other choice experiment applications in the GSE have found strong negative effects of increasing wage and access to microcredits but also of increasing number of cattle on preferences for bushmeat hunting [62].

Overall, almost all respondents prefered expanding existing traditional livelihood strategies focusing on farming (99.97%) and cattle production (71.98%). An evaluation of the implications of these preferences can be made based on the current average area of cropland (6.99 acres ±1.69, 95% CI) and number of cattle owned (16.58 head ± 3.13, 95% CI) per household and assuming that household will convert 22% additional land to cropland and increase the number of cattle owned by 12% based on the estimated average of the levels for these attributes in the choice experiment, excluding negative change for cattle. This rough evaluation suggests that the area of land cultivated will increase by 1.54 acre per household and the number of cattle by 1.43 if travel time to market is reduced by 27 minutes (percentage changes are based on the estimated average positive level of the attributes in the choice experiment). Hence, considering the prevailing land scarcity in large parts of the ecosystem [76] and high human and livestock population growth rates [53], encroachment and illegal grazing in the protected areas may become the dominant future conservation concern rather than bushmeat hunting if roads are improved.

### 4.2. Managing adverse side effects of roads

Little information is available about how incentives can be modified to reduce land use conversion, overstocking and illegal grazing inside protected areas, likely to become increasingly problematic as infrastructure develops in the GSE. We found that provision of low-interest loans had significant negative modifying effects on preferences for expansion of traditional livelihood activities indicating that capital is a barrier to intensification rather than extensification. Similarly, crop extension services had negative modifying effects on preference for expansion of crop production indicating that households would like to intensify crop production (i.e. supporting Hypothesis 1) whereas livestock extension service had a positive modifying effect on preference for expansion of livestock production indicating that households does not favour intensification of livestock production (i.e. contradiciting Hypothesis 1). These results suggests that providing people access to crop extension service such as seeds of improved crop varieties may reduce the additional area converted to cropland while the provision of livestock extension service including improved livestock breeds and veterinary services may increase investment in cattle and the number of cattle added. The same outcome is reflected in the analysis of MRS. The trade-offs with loans, extension services and business and wage employment also show that provision of loans and promoting opportunities for wage employment are potential policy tools to reduce both demand for additional cropland and preference for investing in more cattle. Complementing such intervention with targeted efforts to increase the educational level and capacity for business development (including tourism-oriented businesses) in the GSE could promote engagement in the labour market and development of MSMEs diverting focus from farming and livestock production and hence reduce pressure on the ecosystem. Increasing the education level as well as entrepreneurship spirit across the GSE is a costly long-term, but perhaps cost-effective, investment. More readily available measures may involve improved land use planning with tighter control of land use conversion in critical areas and enforcing existing ceilings on the allowed number of livestock owned. Recently efforts have been made to enforce existing ceilings of a maximum of 200 heads of livestock per household and confiscation and auctioning off cattle caught inside protected areas. Wheather this has a longterm sustainable effect on encroachment remains to be seen.

A considerable number of studies have focused on strategies to reduce illegal hunting and demand for bushmeat in the GSE. Research in western Serengeti concludes that community microcredit programs known as Community Conservation Banks (COCOBAs), providing household level incentives through loans to support the development of alternative livelihood strategies, with the requirement that loan group members abolish bushmeat hunting, has better potential to reduce bushmeat hunting than either TANAPAs Community Conservation Services (CCS) program or WMAs alone ([75], see also [50,77]). Another often suggested solution is improved law enforcement [78]. A study found high aversion towards the risk of being caught hunting illegally in Western Serengeti with a negative preference for hunting trips of one week's duration as long as there was a chance of being caught [62]. However, this result conflicts with the continued occurrence of poaching in the GSE and may be explained by the composition of the sample consisting mainly of non-hunters (see [61]). An increase in the availability of alternative income generating opportunities may more consistently increase the opportunity costs of hunting [61,62]. However, the number of jobs required is substantial [62] as also indicated by our results (1≈2.5 hunting vs employed household members). On the demand side, evidence indicates that bushmeat consumption decline if the price increase relative to its substitutes suggesting that if enforcement can sufficiently increase the cost of supplying and hence the price of bushmeat, then demand will decrease [63,79,80]. However, [79] also found positive wealth elasticity of bushmeat consumption indicating that demand will increase with efforts to increase welfare and household income across the GSE. The study also points to significant interdependencies with other sectors including that substitution away from bushmeat would likely lead to depletion of Lake Victoria fisheries. Similar connections have been highlighted in West Africa [81]. The preference for more cattle observed in this study would likely reduce the price of beef and hence contribute to reducing demand for bushmeat but would have collateral environmental costs through the need for additional grazing land (cf. above).

### 4.3. Targeting incentives

Insights on geographic and between household heterogeneity in the sample can assist in targeting interventions based on the results. Exploring differences between ethnic groups reveal that increasing the number of cattle as travel time is reduced is strongly preferred by Masai pastoralists inhabiting Ngorongoro district along with livestock extension service provision. Hence, efforts to mitigate associated problems may be needed more in this part of the ecosystem where livestock numbers per AEU (Appendix G in S File), although not per square kilometre, are already highest. Preference for increasing cropland was highest among the Kuria agriculturalist inhabiting Tarime district who on the other hand had the lowest preference for increased number of cattle. This suggests a strong need for land use planning in this part of the ecosystem to accommodate effects of infrastructure development. Unlike the former tribes, the Sukuma agro-pastoralists of Bariadi and Meatu districts had the strongest preference for increasing wage employment and aversion towards decreasing hunting and trading bushmeat suggesting that efforts to control possible effects of infrastructure development on bushmeat hunting should focus here and involve alternative income generating opportunities. These differences reflect cultural and livelihood strategy differences between ethnic groups and locations and appropriately incorporating these in conservation policy making is crusial [82]. Distance to protected area boundaries also matters, reducing preferences for increasing effort hunting and trading bushmeat but also for business and wage employment. Evaluating the implications of wealth rank and asset endowments further underline how interventions can be targeted.

### 4.4. Assessment of the empirical model

We found substantial heterogeneity in the estimated effects, indicating that preferences differ considerably between people, which may be explained by the broad geographical extent of our study area. The RPL also had relatively low explanatory power in terms of pseudo R-squared indicating that a large part of the decision reasoning is unobserved. This is likely due to the approach asking about *ex-ante* preferences for decisions with long-lasting effects where options for livelihood change may, in reality, be limited by many other factors than travel time to nearest district town. Such considerations may make respondents uncertain about their preferences. Despite the low explanatory power of the model, the likelihood ratio test suggests that the included attributes significantly improved the model (Chi-squared = 61.89; P<0.05) and many of the attributes were statistically significant meaning that we can draw meaningful conclusions about how changes in significant attributes are associated with changes in utility, which is the primary objective of the current paper.

We asked people to state a priori expectations of livelihood activity change in response to construction or upgrading of roads to reveal what trade-offs they are willing to make. This is a hypothetical question, involving uncertainty about options available as well as uncertainty about own preferences. Hence, the results do not predict what people will actually do but instead reflect what they intend to do. Furthermore, individuals participating in stated preference studies may have incentives to provide strategic answers. Assuming that most respondents are positive towards road development, and they think that the results of the study will affect political decisions they may over-emphasise social desirable livelihood activities relative to illegal activities. However, previous applications of choice experiments in the context of bushmeat hunting have revealed a large potential to provide information about such sensitive activities. Furthermore, the choice experiment was implemented after three or more visits to these household over the course of nine months establishing good rapport with respondents, emphasising that they were anonymous, and answering hypothetical questions and pointing out and explaining the attribute non-attendance feature of the survey, which we believe eliminates any incentives for strategic response in relation to hunting and trading bushmeat. Furthermore, our results show a significant preference for decreased and not “no change” in effort hunting and trading bushmeat (Table 3 and Appendix J in S1 File), which might be the choice if one were concerned about admitting to this illegal activity. Therefore we do not expect the strategic element to be driving the results.

## 5. Conclusions

We assessed support for four hypotheses with diverging effects of road construction and upgrading and opposing conservation implication in the GSE based on choice experiments revealing respondents’ preferences and trade-offs among livelihoods activities. Overall almost all respondents prefered extensification existing traditional livelihood strategies converting more land to cropland and a larger increase in the number of cattle with reduced travel time. Hence, the analysis provides strongest support for Hypothesis 3 indicating that respondents believe that improved access to markets will enable them to market their crop and livestock products more profitably and that they as a result plan extensification of these means of production. This expansion is traded-off against household members allocated to wage employment and involvement in MSMEs and to hunting and trading bushmeat for which there is no preference for increase (contrary to Hypothesis 2 and 4). The lack of preference for engagement in employment and business markets is surprising, and the observed aversion towards reducing the number of household members engaged in hunting suggests that second order outcomes may yet lean towards the scenario in Hypothesis 4. The strong preferences for more cropland and cattle may furthermore interact to increase ecosystem pressure through land use change, overstocking and illegal grazing inside protected areas. Provision of low-interest loans and extension services may modify preferences to reduce the impetus for land conversion and increased livestock production (driving towards Hypothesis 1) but only to a limited extent. Education programs and strategic efforts to facilitate the development of MSMEs that are aligned with conservation goals and take advantage of the large tourism income potential in the GSE may have larger effect and may ultimately provide other means and more profitable investment options than cattle.

## Supporting information

S1 File (Appendix A) Poverty Environment Network survey. (Appendix B) Choice experiment design for the pilot survey. (Appendix C) Choice experiment description, including cheap talk. (Appendix D) The random utility framework. (Appendix E) Description of the socioeconomic covariates included to explain heterogeneity in households preference for different attributes. (Appendix F) Mean coefficient for the random attributes by district. (Appendix G) Mean values of the selected socioeconomic covariates by district. (Appendix H) Random parameter logit model results 1. (Appendix I) Random parameter logit model results 2. (Appendix J) Random parameter logit model results 3.
(DOCX)
