# Supplementary material for: Roads and livelihood activity choices in the Greater Serengeti Ecosystem, Tanzania
Source: PLoS One. 2019 Mar 8;14(3):e0213089. doi: 10.1371/journal.pone.0213089 (PMC6407761; doi:10.1371/journal.pone.0213089)
Supplement: S1 File — (Appendix B) Choice experiment design for the pilot survey. (Appendix C) Choice experiment description, including cheap talk. (Appendix D) The random utility framework. (Appendix E) Description of the socioeconomic covariates included to explain heterogeneity in households preference for different attributes. (Appendix F) Mean coefficient for the random attributes by district. (Appendix G) Mean values of the selected socioeconomic covariates by district. (Appendix H) Random parameter logit model results 1. (Appendix I) Random parameter logit model results 2. (Appendix J) Random parameter logit model results 3. (DOCX) [file pone.0213089.s001.docx]

# **Supplementary materials**

## **Appendix A**

The Poverty Environment Network (PEN) survey is the largest global survey of the importance of environmental goods and services in the livelihoods of forest adjacent rural communities in the Global South to date. The PEN survey employed a socio-economic survey questionnaire that has been implemented in more than 24 tropical countries to understand the relationship between poverty and the environment. The questionnaire is available in eight languages at <https://www.cifor.org/pen/the-pen-prototype-questionnaire/>) and accompanied by a set of technical guideline that describes the elements of the questionnaire and the definitions used. The technical guideline is available in two languages at <https://www.cifor.org/pen/the-pen-technical-guidelines/>.

## **Appendix B**

For the pilot survey, we generated eighteen choice sets (in six blocks) using a sequential orthogonal design (achieves orthogonality within alternatives and results in a lower number of choice sets) with an ex-ante d-error of 0.133. The households were presented with two random blocks, a total of six choice cards.

## **Appendix C**

Consider that the government of Tanzania, supported by a major conservation NGO, is considering to invest a large amount of money in improving road connectivity (through construction of new roads and/or upgrading of existing roads) between villages and urban centres in the Serengeti Ecosystem. As a result, you will have access to market centres using public transport and private trucks all year round reducing travel time (hours and minutes) enabling you to transport your crop and livestock products to the market. In addition, the roads will facilitate the establishment of tourist lodges and other tourism-based businesses attracting investors to your area. This will increase opportunities for wage labour for people in your village. It will also make it easier to seek employment in nearby towns. As a part of this development, the government will also arrange access to (i) micro-loans in order to enable you to expand and engage in business activities, (ii) additional areas of land for cultivation to enable you to produce more crops, including cash crops and (iii) provide extension services to improve your crop and cattle productivity.

We will now play a small game that aims to determine how people will prioritise between livelihood activities given the opportunities provided by the improved road network. In this game, you are presented with six choice cards like this [show an example choice card], and in each card, I will ask you to choose between different livelihood scenarios in accordance with what you believe would provide the best overall livelihood for your household. Please consider each card independently from the previous. Each card presents two alternative future livelihood scenarios in addition to your livelihood of today. Each alternative is described by the following six characteristics:

1. Change in number of adults fully engaged in wage-earning activities and business related activities (e.g., petty trading, transport) [-1, 0, 1]
2. Additional area of land that will be allocated to crop (and cash crop) production including through conversion of land that is currently under other use (in percentages, relative to today) [0%, 10%, 50%]
3. Change in the number of cattle owned (in percentages, relative to today) [-50%, 0%, 25%]
4. Provision of timely extension services (e.g. provision of improved seeds or cows, and veterinary services) [no provision of extension services, extension services for crop production, extension services for livestock production]
5. Household member's engagement in bushmeat hunting and/or bushmeat trade relative to today [increase, no change, decrease]
6. Size of a loan (in Tanzanian shillings) to be repaid in a year in four instalments with 10% interest rate [0, 50000, 200000, 500000, 1000000, 3000000].

[Show the video]

Now it is your turn to make choices, selecting the alternative that is best for your household's overall livelihood as shown in the example. We would like you to think carefully about your choices and consider the likely consequences of these choices for your household's overall wellbeing exactly as if they were real life choices and take note that choosing one strategy means that you would have fewer resources available to adopt or continue other livelihood activities simultaneously. Feel free not to consider any characteristics that are not relevant in your current and future livelihood strategy choices. Notice that there are no right or wrong answers. We are simply interested in your opinion. The answers you provide will be kept confidential.

## **Appendix D**

The theoretical foundation for analysing choice experiment data is the random utility theory. The random utility theory assumes economic rationality and utility maximisation meaning that the household chooses the alternative that yields its highest household benefit, i.e. utility [1]. The utility is assumed to be determined by attributes and their levels constituting the chosen alternative (1). Mathematically, the utility of a household from an alternative can be defined as:

$U_{qi}=X_{q}\beta_{q}+\varepsilon_{q}$ (1)

Where $U_{qi}$ is the utility of household $i$ drives from choosing an alternative $q$, $X_{q}$ is the vector of attributes for alternative $q$ and $\beta_{q}$ are the associated vector of coefficients reflecting the relative contribution of attributes to the utility associated with the choice of alternative and these constitutes the determinist part of the random utility model. $\varepsilon_{q}$ is the models error term and constitutes the stochastic part of the model. The probability that the household prefers alternative $q$ over $j$ in a given choice set is presented by the probability that the utility associated with choosing alternative$q$ is greater than the utility associated with choosing alternative$j$:

$P\left[(U_{qi}>U_{ij})\forall q\#j\right]=P(X_{q}B_{q}-X_{h}B_{h})>(\varepsilon_{q}-\varepsilon_{h})$ (2)

To derive parameter estimates, we make a common assumption about the distribution of the error terms ($\varepsilon_{q}$and $\varepsilon_{h}$). The difference between assumptions leads to a number of modelling alternatives, namely: multinomial logit model, the generalised extreme value models (e.g. nested logit model, paired combinatorial logit model and generalised nested logit model), multinomial probit model, latent class model and random parameters logit model. Although the multinomial logit model is often used due to its convenience, it assumes that the error terms are IID (identically independently distributed) extreme value, which is often inappropriate. The generalised extreme value models cannot capture random variation in error terms and are inconvenient when the data has a panel structure. The multinomial probit model assumes the error terms are jointly normally distributed, which requires solving the probability densities through simulation. This is computationally demanding and often fails to converge. And, the assumption of normal distribution can be inappropriate and result in unreasonable predictions [2].

## **Appendix E**

| Covariate | Type | Description |
| --- | --- | --- |
| Household wealth status | Categorical | Households wealth raking based on village level participatory wealth ranking (1=poor, 2=medium and 3=rich) |
| Head tribe | Categorical | The tribe of the head of the household (1=Massai, 2=Sukuma, 3=Kuria and 9=other (a tribe other than the three mentioned here) |
| Total implements: Value | Continuous | Purchasing power parity (PPP) adjusted total value of implements (e.g. agricultural implements, household utensils, television) owned by the households. |
| Total livestock: TLU* | Continuous | Total cattle owned by the household in Tropical Livestock Units |
| Total cropland: Acre | Continuous | Total cropland owned by the household in Acres |
| Total grazing land: Acre | Continuous | Total grazing land owned by the household in Acres |
| Distance to PA: KM | Continuous | Euclidean distance between households location and the boundary of the nearest protected area in kilometres |

*TLU conversion factors: bull=1.2 TLU, cow=1 TLU, Calf=0.405TLU, sheep/goat=0.2TLU, doe/buck/lamb=0.08 TLU, donkeys=0.8 TLU, pig=0.3 TLU, chicken/birds=0.04TLU

## **Appendix F**

| District | Meatu | Bariadi | Serengeti | Tarime | Ngorongoro | Overall |
| --- | --- | --- | --- | --- | --- | --- |
| Bushmeat hunting and trading decrease | 0.0056 | -0.0085 | 0.0125 | -0.0182 | 0.0156 | 0.0052 |
| Bushmeat hunting and trading increase | 0.0002 | -0.0010 | -0.0055 | -0.0018 | -0.0119 | -0.0049 |
| Increase in crop land | 0.1356 | 0.1438 | 0.1525 | 0.1562 | 0.1639 | 0.1516 |
| change in cattle | 0.0366 | 0.0318 | 0.0444 | 0.0261 | 0.0646 | 0.0438 |
| Wage employment decrease | -0.0187 | -0.0232 | -0.0179 | -0.0282 | -0.0203 | -0.0207 |
| Wage employment increase | 0.0178 | 0.0223 | -0.0007 | 0.0261 | -0.0226 | 0.0039 |
| Livestock extension service | 0.0097 | 0.0030 | 0.0425 | -0.0003 | 0.0831 | 0.0355 |
| Crop extension service | 0.0102 | 0.0041 | 0.0342 | 0.0004 | 0.0697 | 0.0301 |
| Ratio between crop land and cattle | 3.7015 | 4.5272 | 3.4367 | 5.9881 | 2.5368 | 3.4586 |
| Ratio between crop and livestock extension service | 1.0517 | 1.3691 | 0.8055 | -1.3741 | 0.8392 | 0.8459 |

## **Appendix G**

|  | Meatu | Bariadi | Serengeti | Tarime | Ngorongoro | Overall |
| --- | --- | --- | --- | --- | --- | --- |
| Household wealth ranking: Poor | 0.42  (0.01) | 0.41  (0.02) | 0.32  (0.01) | 0.33  (0.01) | 0.34  (0.01) | 0.36  (0.01) |
| Household wealth ranking: Intermediate | 0.36  (0.01) | 0.43  (0.02) | 0.45  (0.01) | 0.49  (0.02) | 0.44  (0.01) | 0.43  (0.01) |
| Household wealth ranking: Rich | 0.22  (0.01) | 0.16  (0.01) | 0.23  (0.01) | 0.18  (0.01) | 0.22  (0.01) | 0.21  (0.00) |
| Head tribe: Massai | 0.00  (0.00) | 0.00  (0.00) | 0.00  (0.00) | 0.00  (0.00) | 0.87  (0.01) | 0.22  (0.00) |
| Head tribe: Sukuma | 1.00  (0.00) | 1.00  (0.00) | 0.12  (0.01) | 0.02  (0.00) | 0.00  (0.00) | 0.34  (0.01) |
| Head tribe: Kuria | 0.00  (0.00) | 0.00  (0.00) | 0.34  (0.01) | 0.93  (0.01) | 0.00  (0.00) | 0.24  (0.01) |
| Head tribe: Other | 0.00  (0.00) | 0.00  (0.00) | 0.54  (0.01) | 0.05  (0.01) | 0.13  (0.01) | 0.20  (0.00) |
| Total implements: Value (in TSH, aeu adjusted) | 409.07  (61.45) | 67.08  (3.51) | 148.15  (5.46) | 231.55  (18.32) | 1482.72  (243.63) | 535.44  (61.84) |
| Total cattle owned: TLU (aeu adjusted) | 1.34  (0.07) | 1.06  (0.05) | 2.37  (0.09) | 1.47  (0.07) | 4.74  (0.15) | 2.48  (0.05) |
| Total livestock other than cattle owned: TLU (aeu adjusted) | 0.31  (0.02) | 0.19  (0.01) | 0.63  (0.04) | 0.36  (0.02) | 3.15  (0.13) | 1.10  (0.04) |
| Total crop land: Acre (aeu adjusted) | 2.15  (0.12) | 1.13  (0.05) | 0.98  (0.02) | 0.91  (0.05) | 0.43  (0.02) | 1.09  (0.03) |
| Total grazing land: Acre (aeu adjusted) | 1.08  (0.11) | 0.17  (0.01) | 0.28  (0.02) | 0.38  (0.03) | 1.31  (0.14) | 0.70  (0.04) |
| Distance to border of protected areas: in Meters | 7722.26  (106.12) | 6066.40  (60.64) | 5899.90  (56.36) | 5314.20  (95.98) | 9960.94  (248.86) | 7203.46  (72.27) |

Note: Values in parenthesis are standard errors

## **Appendix H**

|  | Mean coefficients | Standard deviations |
| --- | --- | --- |
| Wage and business employment (decrease by one household member) X travel time reduction | -0.016*  (0.009) | 0.149***  (0.031) |
| Wage and business employment (increase by one households member) X travel time reduction | -0.104***  (0.019) | 0.096***  (0.017) |
| Wage and business employment (increase by one households member) X Loan/1000000 X travel time reduction | 0.046***  (0.015) | - |
| Increase in crop land (percentage/100) X travel time reduction | 0.496***  (0.084) | 0.033**  (0.014) |
| Increase in crop land (percentage/100) X Loan/1000000 X travel time reduction | -0.517***  (0.117) | - |
| Increase in crop land (percentage/100) X Extension services (crop production) X travel time reduction | -0.288***  (0.096) | - |
| Change in cattle ownership (percentage/100) X travel time reduction | 0.081***  (0.018) | 0.130***  (0.022) |
| Change in cattle ownership (percentage/100) X Loan/1000000 X travel time reduction | -0.031**  (0.013) | - |
| Change in cattle ownership (percentage/100) X Extension services (livestock production) X travel time reduction | 0.134*  (0.069) | - |
| Extension services (crop production) X travel time reduction | 0.215***  (0.036) | 0.142  (0.094) |
| Extension services (livestock production) X travel time reduction | 0.195***  (0.032) | 0.050***  (0.012) |
| Decrease in bushmeat hunting and trading effort × travel time reduction | 0.015  (0.063) | 0.145***  (0.022) |
| Increase in bushmeat hunting and trading effort × travel time reduction | 0.010  (0.010) | 0.172***  (0.028) |
| Loan/1000000 X travel time reduction | 0.039***  (0.007) | - |
| ASC: status quo X travel time reduction | -0.014  (0.027) | - |
| Log Likelihood | -1742.3 | |
| McFadden Pseudo R-squared | 10.0 | |
| # of observations | 2286 | |
| # of respondents | 381 | |

Notes: Coefficients show households’ preference for different livelihood activities, incentives and selected interactions between the increase in cropland, change in cattle and increased wage employment with incentive attributes in the context of road connectivity improvement and resulting reduced travel time to district capital. Values in parenthesis are standard errors; ***, ** and * significant at 1%, 5% and 10% level; the model was estimated with 2000 Halton draws.

## **Appendix I**

|  | Mean coefficients | Standard deviations |
| --- | --- | --- |
| Wage and business employment (decrease by one household member) X travel time reduction | -0.027**  0.011 | 0.123***  0.029 |
| Wage and business employment (increase by one households member) X travel time reduction | -0.005  0.010 | 0.077***  0.017 |
| Increase in crop land (percentage/100) X travel time reduction | 0.176***  0.037 | 0.050**  0.020 |
| Change in cattle ownership (percentage/100) X travel time reduction | 0.055***  0.014 | 0.088***  0.015 |
| Extension services (crop production) X travel time reduction | 0.048***  0.016 | 0.165  0.110 |
| Extension services (livestock production) X travel time reduction | 0.055***  0.016 | 0.039***  0.009 |
| Decrease in bushmeat hunting and trading effort × travel time reduction | -1.147X10^-4^  0.064 | 0.119***  0.017 |
| Increase in bushmeat hunting and trading effort × travel time reduction | -0.010**  0.005 | 0.152***  0.022 |
| Loan/1000000 X travel time reduction | 0.023***  0.005 | - |
| *Loan/1000000 X income (ppp adjusted) X travel time reduction* | *-4.123X10^-7^*  *9.024 X10^-7^* | *-* |
| ASC: status quo X travel time reduction | -0.198***  0.026 | - |
| Log Likelihood | -1778.6 | |
| McFadden Pseudo R-squared | 0.081 | |
| # of observations | 2286 | |
| # of respondents | 381 | |

Notes: Coefficients show households’ preference for different livelihood activities, incentives and interaction of loan with income (ppp adjusted) in the context of road connectivity improvement and resulting reduced travel time to district capital. Values in parenthesis are standard errors; ***, ** and * significant at 1%, 5% and 10% level; the model was estimated with 2000 Halton draws

## **Appendix J**

|  | Mean coefficients | Standard deviations |
| --- | --- | --- |
| Wage and business employment (decrease by one household member) X travel time reduction | -0.018**  (0.008) | 0.126***  (0.027) |
| Wage and business employment (increase by one household member) X travel time reduction | -0.007  (0.010) | 0.071***  (0.016) |
| Increase in crop land (percentage/100) X travel time reduction | 0.190***  (0.037) | 0.033**  (0.013) |
| Change in cattle ownership (percentage/100) X travel time reduction | 0.057***  (0.014) | 0.092***  (0.018) |
| Extension services (crop production) X travel time reduction | 0.052***  (0.016) | 0.167*  (0.097) |
| Extension services (livestock production) X travel time reduction | 0.058***  (0.017) | 0.164  (0.106) |
| No change bushmeat hunting and trading effort × travel time reduction | -0.008  (0.067) | 0.121***  (0.018) |
| Increase in bushmeat hunting and trading effort × travel time reduction | -0.017  (0.067) | 0.154***  (0.024) |
| Loan/1000000 X travel time reduction | 0.024***  (0.005) | - |
| ASC: status quo X travel time reduction | -0.196***  (0.027) | - |
| Log Likelihood | -1779.1 | |
| McFadden Pseudo R-squared | 0.081 | |
| # of observations | 2286 | |
| # of respondents | 381 | |

Notes: Coefficients show households’ preference for different livelihood activities, incentives and with no change in bushmeat hunting and trading effort in place of decrease in bushmeat hunting effort in the context of road connectivity improvement and resulting reduced travel time to district capital. Values in parenthesis are standard errors; ***, ** and * significant at 1%, 5% and 10% level; the model was estimated with 2000 Halton draws

# **References cited**

1. Lancaster KJ. A New Approach to Consumer Theory. Journal of Political Economy. 1966;74(2):132–57.

2. Train KE. Discrete choice methods with simulation. Second. New York: Cambridge University Press; 2009.
